# Phylogeography, colouration, and cryptic speciation across the Indo-Pacific in the sea urchin genus *Echinothrix*

**DOI:** 10.1038/s41598-021-95872-0

**Published:** 2021-08-16

**Authors:** Simon E. Coppard, Holly Jessop, Harilaos A. Lessios

**Affiliations:** 1 Smithsonian Tropical Research Institute, Box 0843-03092, Balboa, Panama; 2 Present Address: Bader International Study Centre, Queen’s University (Canada), Herstmonceux Castle, Hailsham, East Sussex, BN27 1RN UK

**Keywords:** Marine biology, Molecular evolution

## Abstract

The sea urchins *Echinothrix calamaris* and *Echinothrix diadema* have sympatric distributions throughout the Indo-Pacific. Diverse colour variation is reported in both species. To reconstruct the phylogeny of the genus and assess gene flow across the Indo-Pacific we sequenced mitochondrial 16S rDNA, ATPase-6, and ATPase-8, and nuclear 28S rDNA and the Calpain-7 intron. Our analyses revealed that *E. diadema* formed a single trans-Indo-Pacific clade, but *E. calamaris* contained three discrete clades. One clade was endemic to the Red Sea and the Gulf of Oman. A second clade occurred from Malaysia in the West to Moorea in the East. A third clade of *E. calamaris* was distributed across the entire Indo-Pacific biogeographic region. A fossil calibrated phylogeny revealed that the ancestor of *E. diadema* diverged from the ancestor of *E. calamaris* ~ 16.8 million years ago (Ma), and that the ancestor of the trans-Indo-Pacific clade and Red Sea and Gulf of Oman clade split from the western and central Pacific clade ~ 9.8 Ma. Time since divergence and genetic distances suggested species level differentiation among clades of *E. calamaris*. Colour variation was extensive in *E. calamaris*, but not clade or locality specific. There was little colour polymorphism in *E. diadema*.

## Introduction

Interpreting phylogeographic patterns of marine species and understanding levels of connectivity among populations across the World’s oceans is of increasing importance for informed conservation decisions^1–3^. The identification of biogeographic barriers that impede dispersal and restrict connectivity among conspecifics can explain levels of genetic isolation^4^. Traditionally species distributions and levels of endemism have been used to define biogeographic regions^5–7^. However, an increasing number of molecular phylogenies have revealed cryptic species (e.g. Coppard et al.^8^; Lemer et al.^9^), requiring continuous modification of designated biogeographic regions based on levels of endemism^10^.

Large distances potentially limit levels of gene flow between populations^11^. Molecular differentiation can be caused by limited genetic exchange between populations, but it can also appear to be so, due to limited sampling^12^. Sampling that covers the entire distribution of widespread marine species can be logistically problematic, but when possible, it can reveal patterns that illuminate the evolutionary history of a taxon. In this study we use high-density sampling to study gene flow and connectivity across the entire Indo-Pacific in a sea urchin genus with a remarkably wide distribution. The two extant species of the genus *Echinothrix* Peters, 1853 are sympatric on coral reefs throughout the Indo-Pacific, central Pacific and eastern Pacific^13^. *Echinothrix calamaris* (Pallas, 1774) and *E. diadema* (Linnaeus, 1758) have planktotrophic larvae. The adults are important herbivores on coral reefs^14–16^. *Echinothrix diadema* maintains on-going gene flow throughout the 5400 km of uninterrupted deep water between the central and the eastern Pacific^17^. As crossing this Eastern Pacific Barrier (EPB) between Kiribati and Clipperton Island is virtually impossible for most shallow water marine invertebrates^18–21^, the ability of *E. diadema* to do so suggests high dispersal potential.

The second species in this genus, *E. calamaris*, displays extraordinary variation in colour throughout its distribution^13,22–24^. The test epidermis and the spines of *E. calamaris* vary from white, to green, red, purple, brown to black, with, or without spots of different colour on the periproctal cone, and often with banding on the interambulacral spines that can be green, brown, purple or red^13^. *Echinothrix diadema* is typically darker and more uniform in colour than *E. calamaris*. Colour polymorphism can play an important role in an individual’s fitness through predator avoidance via camouflage^25^, or aposematic colouring and protection against ultraviolet light^26^. Regional variations in diet or responses to environmental conditions (water depth, distribution/exposure to UV light, water temperature and predation pressure) may favour an increased prevalence of particular colour morphs. Brighter colours may ward off predators and warn about the venomous nature of a sea urchin’s ambulacral spines^27,28^; green or brown colours that confer a degree of camouflage in seaweeds may reduce predation pressure by visual predators. Colour variation may also correlate with genetic divergence and possible speciation. Mortensen^13^ suggested that regional colour differences existed in *E. calamaris*, with darker colour morphs prevailing in the western part of the Indian Ocean and lighter colour morphs being most common in the Malay Archipelago.

Species of *Echinothrix* have a poor fossil record primarily due to the fragile nature of their tests that break-up and disperse after death. Spines of this genus are preserved but are often broken into small fragments less than 10 mm long^29^. A spine fragment from Java, Indonesia (Fig. 2f. in Ref.^29^) is typical of *E. calamaris* with tight, non-flaring verticillations (see Coppard & Campbell^28^). This spine fragment dates from the Burdigalian 20.44–15.97 million years ago (Ma) placing *Echinothrix,* or its ancestor, in the Indian Ocean during the Early Miocene.

In this study, we combine mitochondrial (mtDNA) and nuclear (nDNA) gene sequences to reconstruct the phylogeny of *Echinothrix* and use it to determine timing of divergence. We assess gene flow between populations in *E. calamaris* and *E. diadema* throughout the Indo-Pacific and the efficacy of geographic barriers. Finally, we address the question of whether there is any genetic structure reflected by clade-specific colouration.

## Results

### Phylogeography

**Figure 1:**
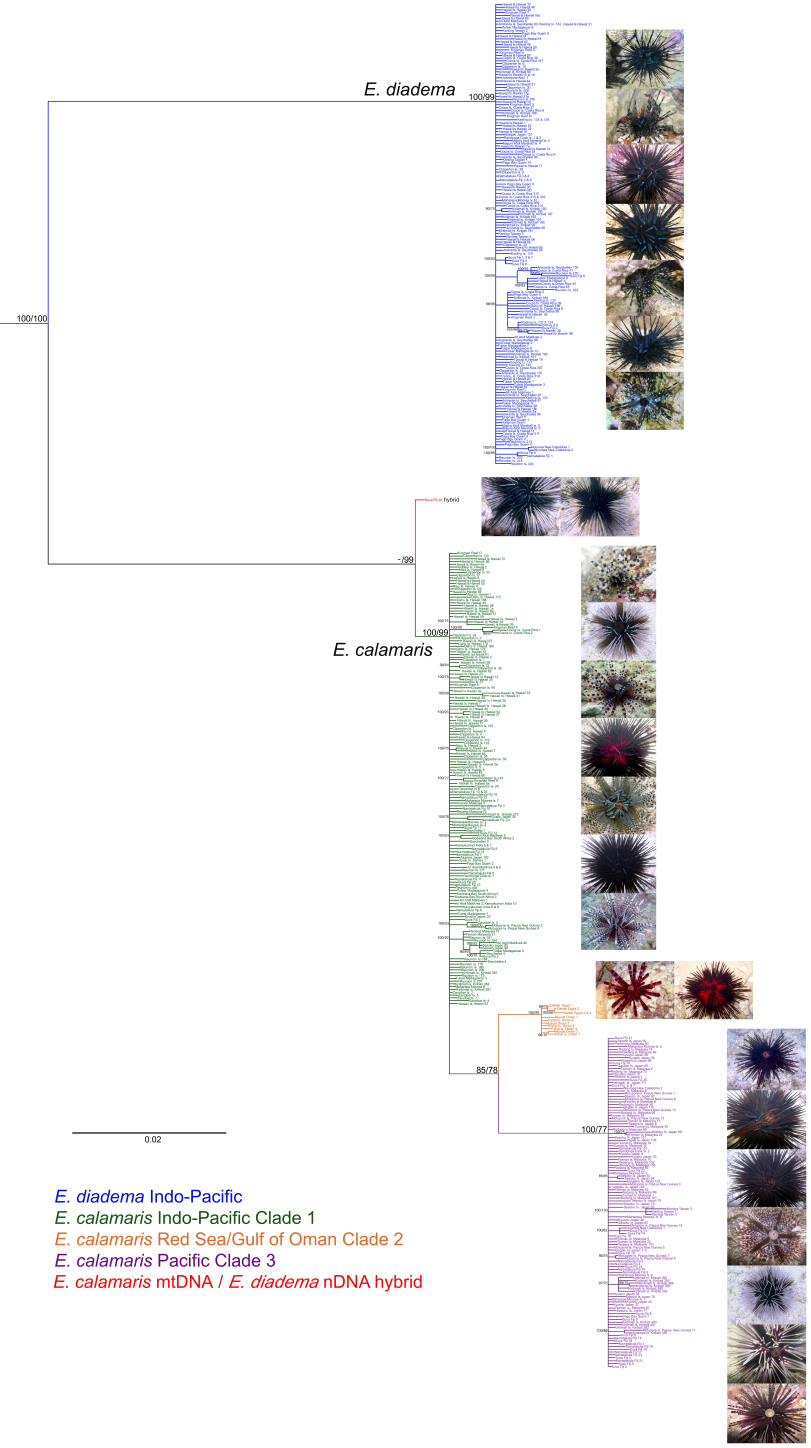
Phylogeny of *Echinothrix* based on concatenated 16S, ATPase-6, ATPase-8, 28S and Calpain-7 intron sequences reconstructed with RAxML, rooted on *Astropyga pulvinata*. Clade credibility values > 85% of Bayesian (first number next to node) and > 75% of Maximum Likelihood (second number) reconstruction are shown, –: < 75%. Scale bar reflects number of changes per site, photos show colour morphs present in each molecular clade.

Bayesian Inference (BI) and Maximum Likelihood (ML) produced congruent phylogenies, with only slight variations in weakly supported clades (Fig. 1). Phylogenies conflicted only in the position of a single hybrid from Suva, Fiji (discussed below), which had mtDNA of *E. calamaris* and nDNA of *E. diadema.* The phylogeny, rooted on *Astropyga pulvinata* (Lamarck, 1816)*,* revealed that sequences of *Echinothrix diadema* formed a single clade, while those of *E. calamaris* contained three divergent clades. ML resolved the concatenated sequence of the hybrid specimen as sister to all *E. calamaris* (Fig. 1), whereas BI placed the sequence of the hybrid individual as sister to clade 3 of *E. calamaris*, reflecting its mtDNA haplotype.

**Figure 2:**
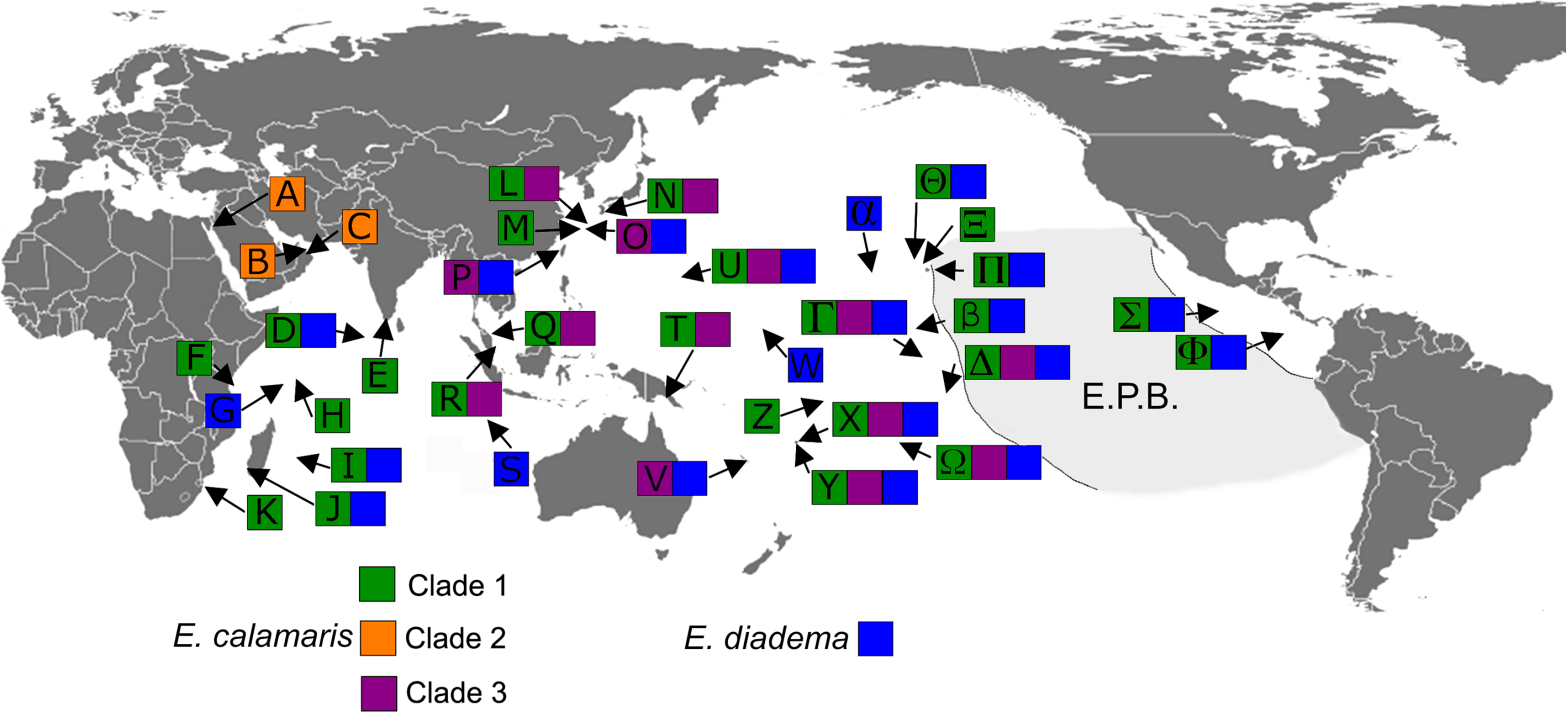
Distribution of clades of *Echinothrix*. Letter refers to locality, colour to the clade. E.P.B. = Eastern Pacific Barrier. A: Dahab, Egypt, Gulf of Aqaba (28.5236° N, 34.5301° E); B: Muscat, Oman, Gulf of Oman (23.9871° N, 58.1859° E); C: Al Fahal Island, Gulf of Oman (23.6822° N, 58.5020° E); D: Ari Atoll, Maldives (3.8825° N, 72.8340° E); E: Kanyakumari, India (8.0798° N, 77.5276° E); F: Zanzibar Island, Tanzania (6.1374° S, 39.1684° E); G: Amirante Islands, Seychelles (6.1554° S, 52.8513° E); H: Seychelles (4.7178° S, 55.4260° E); I: Réunion Island (21.0271° S, 55.7966° E); J: Tuléar, Madagascar (23.3833° S, 43.6667° E); K: Sodwana Bay, South Africa (27.5187° S, 32.6915° E); L: Sesoko Island, Japan (26.6333° N, 127.8667° E); M: Akajima Island, Japan (26.2042° N, 127.2876° E); N: Kyushu, Japan (31.0553° N, 130.3640° E); O: Ishigaki Island, Japan (24.46307° N, 124.2747° E); P: Kenting, Taiwan (21.9447° N, 120.7750° E); Q: Redang Island, Malaysia (5.7893° N, 103.0184° E); R: Tioman Island, Malaysia (2.8162° N, 104.1543° E); S: Cocos (Keeling) Islands (12.1889° S, 96.8197° E); T: Motupore Island, Papua New Guinea (9.5233° S, 147.2860° E); U: Pago Bay, Guam (13.4207° N, 144.7940° E); V: Noumea, New Caledonia (22.2959° S, 166.4370° E); W: Majuro Atoll, Marshall Islands (7.0915° N, 171.3772° E); X: Suva Lagoon, Fiji (18.1777° S, 178.4875° E); Y: Namatakula, Fiji (18.2372° S, 177.7845° E); Z: Upola Island, Samoa (13.7835° S, 171.8589° W); α: Johnston Atoll (16.7405° N, 169.5074° W); β: Kingman Reef (6.4188° N, 162.3655° W); Ω: Rarotonga, Cook Islands (21.2142° S, 159.7126° W); Δ: Maharepa, Moorea (17.4758° S, 149.7929° W); Γ: Kiritimati Island, Kiribati (Line Islands) (1.9213° N, 157.4442° W); Θ: Oahu, Hawaii (21.2951° N, 157.8830° W); Π: Hawaii Island, Hawaii (19.7308° N, 154.9915° W); Ξ: Maui, Hawaii (20.6267° N, 156.1925° W); Σ: Clipperton Island (10.2862° N, 109.2124° W); Φ: Isla del Coco, Costa Rica (5.5360° N, 87.0244° W). The outline of this map was downloaded from Dmthoth, CC BY-SA 3.0, via Wikimedia Commons and edited in Photoshop Elements 2020.

Remarkably, *E. diadema* and clade 1 of *E. calamaris* had geographic distributions that stretched the length and breadth of the tropical Indo-Pacific (Fig. 2.), from South Africa and the Seychelles in the West to Japan in the North, New Caledonia in the South and Isla del Coco and Clipperton Island in the East. Clade 2 was restricted to the Red Sea and the Gulf of Oman. Clade 3 was distributed in the Pacific from Malaysia in the West, to Japan in the North, New Caledonia in the South, and Moorea in the East. Members of this clade were not present in the Hawaiian Archipelago or at Kingman Reef.

Genetic distances in ATPase-6 and ATPase-8 between clades within *E. calamaris* were similar to those between *E. calamaris* and *E. diadema* suggesting species level differentiation. However, genetic distances in 16S, 28S and the Calpain-7 intron were substantially smaller between clades of *E. calamaris* than between *E. calamaris* and *E. diadema* (Table 1).

**Table 1:** Mean genetic difference between DNA sequences of *Echinothrix diadema* (*E. d.*) and clades of *Echinothrix calamaris* (*E. c.*) calculated using models suggested by jMODELTEST for each gene.

Species/clade	16S (%)	ATPase6 (%)	ATPase8 (%)	28S (%)	Calpain-7 intron (%)
*E. d.* versus *E. c.* clade 1 (IP)	12.75	5.15	4.67	0.25	10.44
*E. d.* versus *E. c.* clade 2 (RS + GO)	13.78	5.10	4.78	0.26	9.82
*E. d.* versus *E. c.* clade 3 (P)	12.84	5.34	4.42	0.25	10.33
*E. c.* clade 1 (IP) versus *E. c.* clade 2 (RS + GO)	2.56	3.49	2.71	0.09	1.55
*E. c.* clade 1 (IP) versus *E. c.* clade 3 (P)	2.82	7.37	5.96	0.09	2.37
*E. c.* clade 2 (RS + GO) versus *E. c.* clade 3 (P)	3.05	6.39	4.95	0.06	1.47

See Fig. 1 for clade designations.

*RS* Red Sea, *GO* Gulf of Oman, *IP* Indo-Pacific, *P* Pacific.

### Timing of divergence

**Figure 3:**
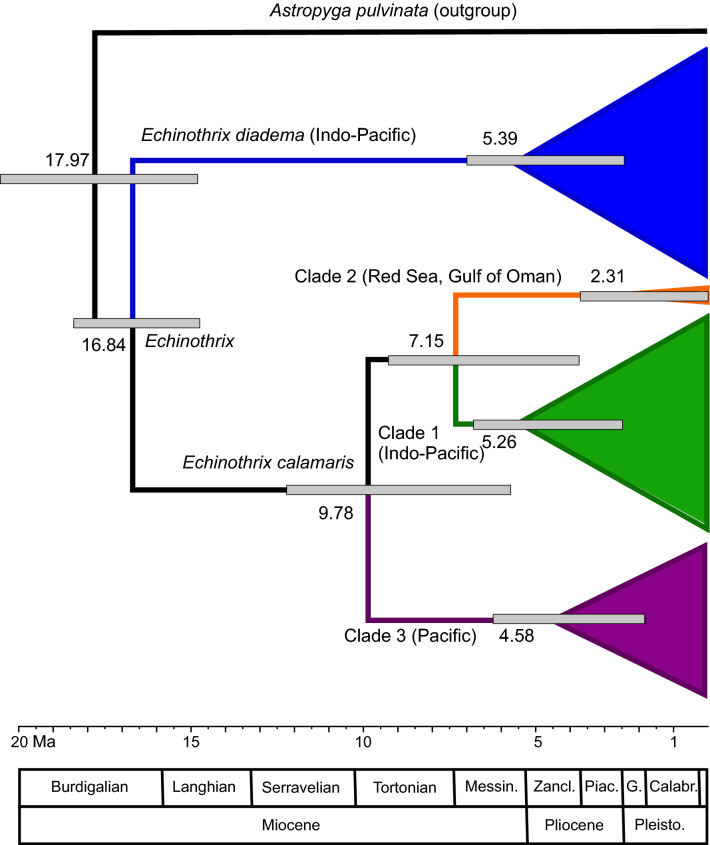
Bayesian estimates of median molecular divergence times (median dates next to node) based on concatenated ATPase-6 andATPase-8, as derived from analysis using BEAST and calibrated using minimum age of genus and substitution rates from *Diadema*, a confamilial genus. Bars indicate 95% Highest Posterior Density (HPD) limits. Ages of stages and epoch series based on International Commission on Stratigraphy stratigraphic chart v.2020/01^30^ (Messin. = Messinian, Zancl. = Zanclean, Piac. = Piacenzian, G. = Gelasian, Calabr. = Calabrian, Pleisto. = Pleistocene).

*Echinothrix diadema* and *E. calamaris* diverged ~ 16.8 Million years ago (Ma) in the Burdigalian stage of the early Miocene (Fig. 3). *Echinothrix calamaris* then split into two lineages ~ 9.8 Ma, one that today is restricted to the central and western Pacific and another now present in the Indo-Pacific. This Indo-Pacific lineage split into two clades ~ 7.2 Ma in the late Tortonian stage of the Miocene. One clade that is today distributed across the entire Indo-Pacific and a second clade in the Red Sea and Gulf of Oman.

### Mitochondrial DNA gene flow

**Figure 4:**
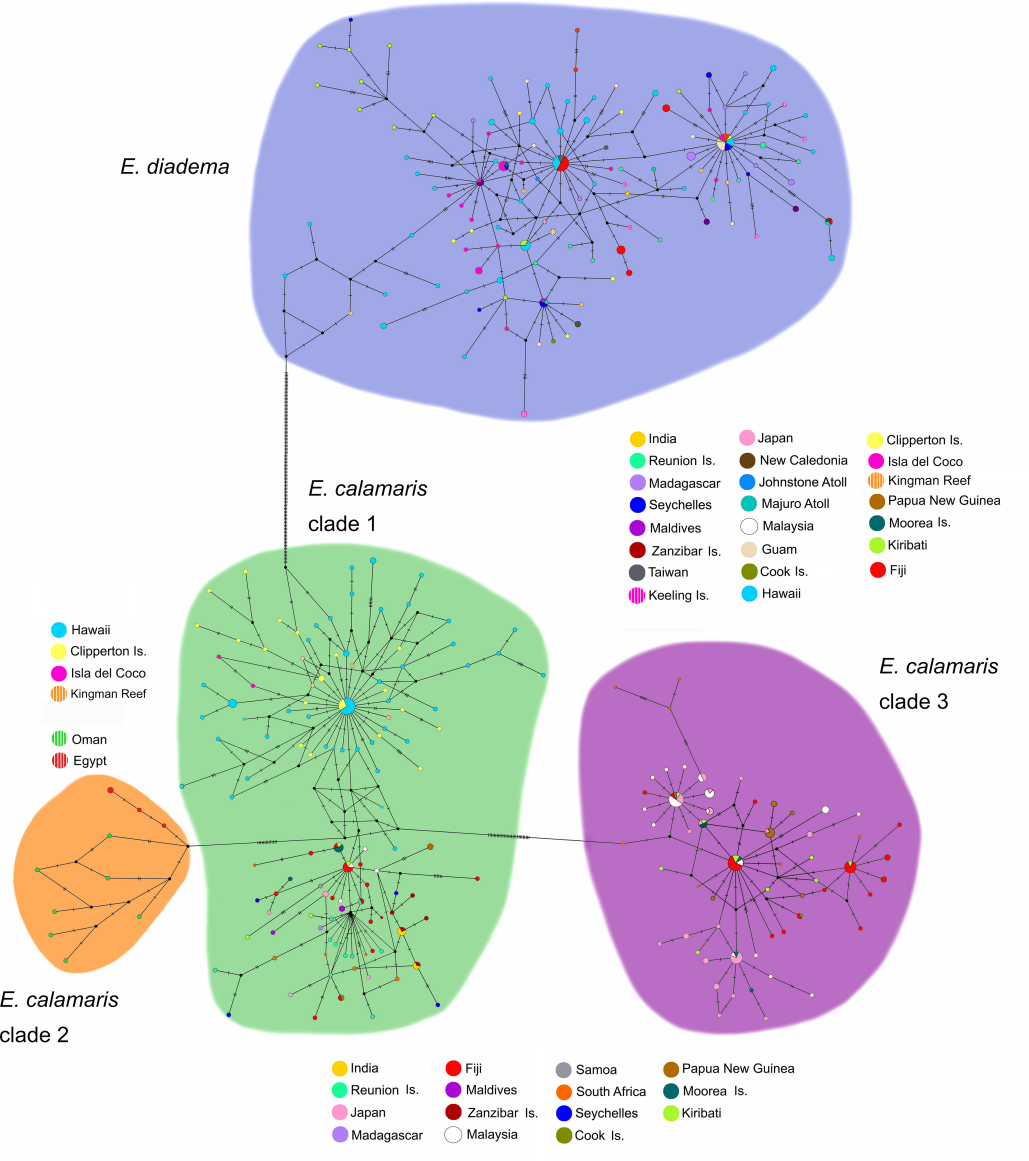
Median joining haplotype network based on mitochondrial ATPase6, ATPase8 and 16S. Constructed in PopART with reticulation tolerance set to zero.

The mtDNA median joining network (Fig. 4) revealed the presence of shared and closely connected haplotypes in populations from Hawaii, Isla del Coco, Kingman Reef and Clipperton Island in clade 1 of *E. calamaris*. These were linked to haplotypes in central, South and western Pacific populations by hypothetical haplotypes that were either not sampled or are extinct. *Φ*_*ST*_ values for mtDNA (Table Supplementary S1) indicated there was gene flow between the Island of Hawaii (Big Island) and Maui in the Hawaiian Archipelago, between the Island of Hawaii and Kingman Reef in the central Pacific, and between Kingman Reef and Clipperton Island in the tropical eastern Pacific. Among the Hawaiian Islands gene flow was restricted between the Island of Hawaii and Oahu (*Φ*_*ST*_ value 0.286, *p* < 0.01), and between Maui and Oahu (*Φ*_*ST*_ value 0.444, *p* < 0.01) (Table S1). Small but significant mtDNA *Φ*_*ST*_ values were found between populations in Maui and Clipperton Island (*Φ*_*ST*_ value 0.069, *p* < 0.05), between Maui and Kingman Reef (*Φ*_*ST*_ value 0.105, *p* < 0.05) and between the Island of Hawaii and Clipperton (*Φ*_*ST*_ value 0.100, *p* < 0.01), suggesting genetic exchange.

In the central Pacific, gene flow of mtDNA in clade 1 of *E. calamaris* was restricted between geographically close populations (~ 760 km) at Kingman Reef and Kiribati (*Φ*_*ST*_ value 0.613, *p* < 0.05). This was also true for populations at Kingman Reef and Moorea (*Φ*_*ST*_ value 0.709, *p* < 0.05), but not between Kiribati and Moorea (Table S1). Gene flow between Kiribati and the majority of other populations was restricted.

Among Indo-West Pacific members of *E. calamaris* clade 1, non-significant mtDNA *Φ*_*ST*_ values indicated the presence of gene flow between Malaysia and Fiji, Japan, Seychelles, Maldives, and South Africa, and between Japan and Moorea, Kiribati, Maldives, Madagascar, and South Africa. Identical haplotypes occurred in populations at Fiji, Moorea, Cook Islands, and Malaysia (Fig. 4).

In the Indian Ocean large and significant *Φ*_*ST*_ values (ranging from 0.34 to 0.56) in the mtDNA of *E. calamaris* clade 1 suggested high levels of genetic isolation of populations at Réunion, with genetic exchange only with Madagascar (Table S1). A degree of isolation was evident in the population off of India. However, *Φ*_*ST*_ values indicated gene flow between the Indian population with the Seychelles, Maldives, and Zanzibar, with which it shared identical mtDNA haplotypes. Connectivity was also found among *E. calamaris* mtDNA haplotypes from India, Zanzibar, and South Africa (Fig. 4).

Populations of *E. calamaris* from the Red Sea and Gulf of Oman in clade 2 were clearly differentiated from each other in mtDNA (*Φ*_*ST*_ value = 0.54, *p* < 0.05) (Table S2).

Populations of *E. calamaris* in clade 3, showed less genetic isolation in mtDNA among populations than those in clade 1. Identical haplotypes were found in individuals at Fiji, Japan, Moorea, Kiribati and Malaysia (Fig. 4), but gene flow between populations in Malaysia or Japan and populations situated farther East in the Pacific was more restricted (Table S3). The population of *E. calamaris* in Papua New Guinea had large and significant *Φ*_*ST*_ values in comparison to all other populations in this clade.

Haplogroups of *E. diadema* included some populations that were distributed far apart in both the Pacific Ocean (Fiji, Moorea, Clipperton, Isla del Coco, Hawaii, Guam) and the Indian Ocean (Seychelles and Zanzibar) (Fig. 4). The presence of shared haplotypes among such disparate populations suggests wide dispersal across the entire Indo-Pacific, including migration across the EPB. Despite many shared and closely connected haplotypes, *Φ*_*ST*_ values indicated genetic heterogeneity among many populations (Table S4). Significant but small *Φ*_*ST*_ values may result from infrequent colonization as suggested by the haplotype network (Fig. 4). Furthermore, *Φ*_*ST*_ values suggested gene flow between Taiwan in the West Pacific and distant populations in the eastern Pacific (Isla del Coco, Clipperton Island) and the Indian Ocean (Seychelles, Keeling Islands).

### Nuclear DNA differentiation

**Figure 5:**
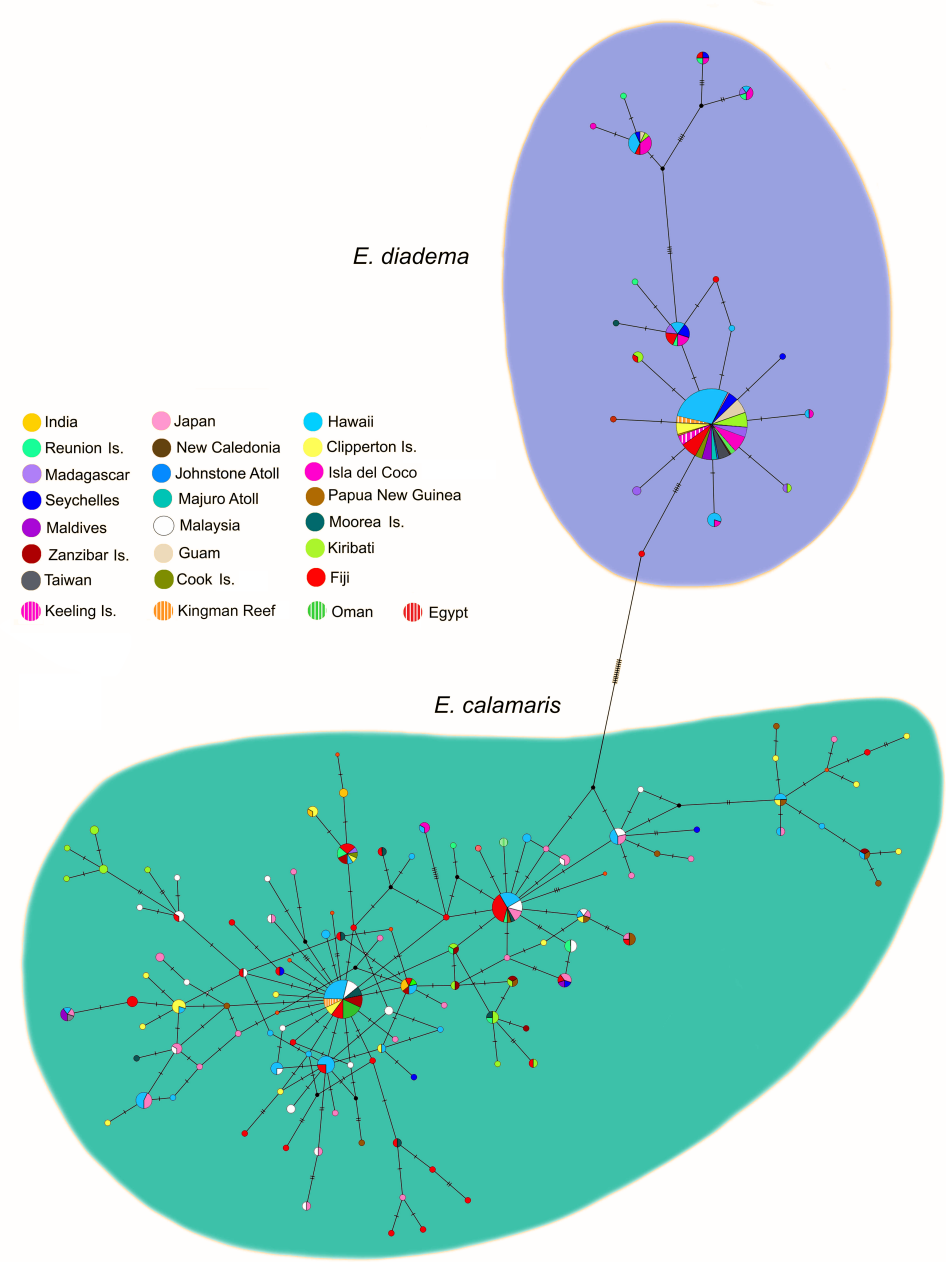
Median joining haplotype network based on 28S rDNA and intron of Calpain-7 nuclear DNA. Constructed in PopART with reticulation tolerance set to zero.

Differences in *Φ*_*ST*_ values between nDNA and mtDNA could be due to sex-specific migration or to the slower rate of evolution due to larger effective population size of nDNA. In clade 1 of *Echinothrix calamaris*, nDNA of the population at Kiribati showed large and significant *Φ*_*ST*_ values (range from 0.24 to 0.44) when compared with Pacific populations at Hawaii, Clipperton, Kingman Reef, Fiji, Japan, or Malaysia, but also small and non-significant *Φ*_*ST*_ values (*p* > 0.05) when compared with some populations in the Indian Ocean (South Africa, Madagascar, Zanzibar, and the Seychelles) (Table S5). *Φ*_*ST*_ values for nDNA in clade 2 of *E. calamaris* suggested connectivity between the Red Sea and Gulf of Oman. In clade 3 of *E calamaris* only populations at Kiribati and Papua New Guinea showed signs of genetic isolation (Table S6). Both *Φ*_*ST*_ values for nDNA (Table S7) and the nDNA haplotype network (Fig. 5) indicated that genetic exchange of populations of *E. diadema* in the Indo-Pacific was far more pervasive than suggested by mtDNA (Table S4). Only a few populations showed indications of restricted gene flow (e.g. Clipperton vs Namatakula, Fiji).

### Genetic differentiation between geographic regions

Analysis of Molecular Variance (AMOVA) of geographic regions within clades of *E. calamaris* and *E. diadema* indicated that most of the genetic variation in both mtDNA and nDNA occurred within populations, rather than between populations within each geographic region or between geographic regions (Tables 2, 3). An exception to this was clade 1 of *E. calamaris*, which showed a greater level of mtDNA variation between geographic regions (32.55%) than between populations within regions (17.39%).

**Table 2:** AMOVA based on mtDNA Tamura and Nei^31^ distances comparing differentiation between and within geographic regions (Indian Ocean, western Pacific Ocean, central Pacific Ocean, Hawaiian Archipelago, tropical eastern Pacific) in each clade of *Echinothrix*.

Clade	Source of variation	Percent variation	Statistics	*p*
*E. calamaris* clade 1	Between regions	32.55	*Φ*_*CT*_ = 0.326	0.007
	Between populations	17.39	*Φ*_*ST*_ = 0.499	< 0.001
	Within populations	50.06	*Φ*_*SC*_ = 0.258	< 0.001
*E. calamaris* clade 3	Between regions	12.09	*Φ*_*CT*_ = 0.121	0.016
	Between populations	14.13	*Φ*_*ST*_ = 0.262	< 0.001
	Within populations	73.77	*Φ*_*SC*_ = 0.161	< 0.001
*E. diadema*	Between regions	-4.72	*Φ*_*CT*_ = -0.047	0.822
	Between populations	20.66	*Φ*_*ST*_ = 0.159	< 0.001
	Within populations	84.06	*Φ*_*SC*_ = 0.197	< 0.001

**Table 3:** AMOVA based on nDNA Tamura and Nei^31^ distances comparing differentiation between and within geographic regions (Indian Ocean, western Pacific Ocean, central Pacific Ocean, Hawaiian Archipelago, tropical eastern Pacific) in each clade of *Echinothrix*.

Clade	Source of variation	Percentage of variation	Statistics	*p*
*E. calamaris* clade 1	Between regions	− 2.82	*Φ*_*CT*_ = − 0.028	0.550
	Between populations	16.07	*Φ*_*ST*_ = 0.132	< 0.001
	Within populations	86.75	*Φ*_*SC*_ = 0.156	< 0.001
*E. calamaris* clade 3	Between regions	2.10	*Φ*_*CT*_ = 0.021	0.029
	Between populations	7.31	*Φ*_*ST*_ = 0.094	< 0.001
	Within populations	90.59	*Φ*_*SC*_ = 0.075	< 0.001
*E. diadema*	Between regions	− 1.62	*Φ*_*CT*_ = − 0.016	0.796
	Between populations	1.79	*Φ*_*ST*_ = 0.002	0.356
	Within populations	99.83	*Φ*_*SC*_ = 0.018	0.318

Pairwise AMOVAs for *E. calamaris* clade 1 demonstrated significant genetic differentiation in mtDNA between populations in the tropical eastern Pacific and those of the Indian Ocean (*Φ*_*CT*_ value = 0.455, *p* = 0.029), but not between the eastern Pacific and populations in the Hawaiian Archipelago *Φ*_*CT*_ value = − 0.051, *p* = 0.489), the central Pacific (*Φ*_*CT*_ value = 0.305, *p* = 0.150) or western Pacific (*Φ*_*CT*_ value = 0.558, *p* = 0.345) (Table 4).

**Table 4:** Between region pairwise AMOVA based on mtDNA Tamura and Nei^31^ distances in *Echinothrix calamaris* clade 1.

	Indian	Ocean	Statistic	*p*	Central	Pacific	Ocean	*p*	Western	Pacific	Ocean	*p*	Tropical	eastern	Pacific	*p*
	S.O.V.	% var.			S.O.V.	% var.	Statistic		S.O.V.	% var.	Statistic		S.O.V.	% var.	Statistic	
Central	B.R.	14.77	*Φ*_*CT*_ = 0.148	0.021												
Pacific	B.P.	29.74	*Φ*_*ST*_ = 0.445	< 0.001												
Ocean	W.P.	55.49	*Φ*_*SC*_ = 0.349	< 0.001												
Western	B.R.	7.45	*Φ*_*CT*_ = 0.074	0.193	B.R.	− 9.84	*Φ*_*CT*_ = − 0.098	0.864								
Pacific	B.P.	28.08	*Φ*_*ST*_ = 0.355	< 0.001	B.P.	32.85	*Φ*_*ST*_ = 0.230	< 0.001								
Ocean	W.P.	64.48	*Φ*_*SC*_ = 0.303	< 0.001	W.P.	76.98	*Φ*_*SC*_ = 0.299	< 0.001								
Tropical	B.R.	45.45	*Φ*_*CT*_ = 0.455	0.029	B.R.	30.34	*Φ*_*CT*_ = 0.305	0.150	B.R.	55.79	*Φ*_*CT*_ = 0.558	0.345				
Eastern	B.P.	16.25	*Φ*_*ST*_ = 0.617	< 0.001	B.P.	23.89	*Φ*_*ST*_ = 0.544	< 0.001	B.P.	− 0.38	*Φ*_*ST*_ = 0.554	< 0.001				
Pacific	W.P.	38.30	*Φ*_*SC*_ = 0.298	< 0.001	W.P.	45.57	*Φ*_*SC*_ = 0.344	< 0.001	W.P.	44.59	*Φ*_*SC*_ = − 0.009	0.326				
Hawaiian	B.R.	46.17	*Φ*_*CT*_ = 0.462	0.007	B.R.	33.60	*Φ*_*CT*_ = 0.336	0.0323	B.R.	45.66	*Φ*_*CT*_ = 0.457	0.104	B.R.	− 5.14	*Φ*_*CT*_ = − 0.051	0.489
Archipelago	B.P.	12.27	*Φ*_*ST*_ = 0.584	< 0.001	B.P.	16.23	*Φ*_*ST*_ = 0.498	< 0.001	B.P.	6.71	*Φ*_*ST*_ = 0.524	< 0.001	B.P.	17.55	*Φ*_*ST*_ = 0.124	< 0.001
	W.P.	41.56	*Φ*_*SC*_ = 0.228	< 0.001	W.P.	50.17	*Φ*_*SC*_ = 0.244	< 0.001	W.P.	47.63	*Φ*_*SC*_ = 0.123	< 0.001	W.P.	87.59	*Φ*_*SC*_ = 0.167	< 0.001

*S.O.V.* source of variation, *B.R.* between regions, *B.P.* between populations within regions, *W.P.* within populations, % *var*. percentage of variation.

*n.b.* Tropical eastern Pacific = Clipperton Island.

The populations in the Hawaiian Archipelago showed significant inter-regional mtDNA differences with those in the Indian Ocean (*Φ*_*CT*_ value = 0.462, *p* = 0.007). They also had a high percentage of significant regional variation with populations in the rest of the central Pacific (33.60%, *Φ*_*CT*_ value = 0.336, *p* = 0.032), but were not significantly different from populations in the western Pacific (*Φ*_*CT*_ value = 0.462, *p* = 0.102). There was no significant genetic variation between populations from the western Pacific and the Indian Oceans (7.45%, *Φ*_*CT*_ value = 0.074, *p* = 0.193), although there were significant mtDNA differences between the inhabitants of the central Pacific and the Indian Ocean (14.77%, *Φ*_*CT*_ value = 0.148, *p* = 0.021)*.*

Analysis of molecular variance of nDNA between regions of *E. calamaris* in clade 1 indicated that most of the genetic variation occurred within populations (Table 5). Only populations in the tropical eastern Pacific and in the Hawaiian Archipelago showed inter-regional variance that slightly exceeded intraregional variance when compared with those from the western Pacific Ocean. However, fixation indices were not significant.

**Table 5:** Between region pairwise AMOVA of nDNA Tamura & Nei^31^ distances in *Echinothrix calamaris* clade 1.

	India	Ocean	Statistic	*p*	Central	Pacific	Ocean	*p*	Western	Pacific	Ocean	*p*	Tropical	eastern	Pacific	*p*
	S.O.V.	% var.			S.O.V.	% var.	Statistic		S.O.V.	% var.	Statistic		S.O.V.	% var.	Statistic	
Central	B.R.	− 3.25	*Φ*_*CT*_ = − 0.032	0.813												
Pacific	B.P.	18.32	*Φ*_*ST*_ = 0.151	< 0.001												
Ocean	W.P.	84.92	*Φ*_*SC*_ = 0.177	< 0.001												
Western	B.R.	0.75	*Φ*_*CT*_ = 0.007	0.413	B.R.	− 0.49	*Φ*_*CT*_ = 0.005	0.576								
Pacific	B.P.	14.62	*Φ*_*ST*_ = 0.154	< 0.001	B.P.	17.08	*Φ*_*ST*_ = 0.166	< 0.001								
Ocean	W.P.	84.63	*Φ*_*SC*_ = 0.147	0.002	W.P.	83.38	*Φ*_*SC*_ = 0.170	< 0.001								
Tropical	B.R.	− 3.33	*Φ*_*CT*_ = − 0.033	0.746	B.R.	− 9.55	*Φ*_*CT*_ = − 0.095	0.846	B.R.	6.70	*Φ*_*CT*_ = 0.067	0.341				
Eastern	B.P.	16.27	*Φ*_*ST*_ = 0.129	< 0.001	B.P.	21.29	*Φ*_*ST*_ = 0.117	< 0.001	B.P.	4.58	*Φ*_*ST*_ = 0.113	0.032				
Pacific	W.P.	87.06	*Φ*_*SC*_ = 0.157	< 0.001	W.P.	88.27	*Φ*_*SC*_ = 0.194	< 0.001	W.P.	88.73	*Φ*_*SC*_ = 0.049	0.277				
Hawaiian	B.R.	2.41	*Φ*_*CT*_ = 0.024	0.215	B.R.	− 4.06	*Φ*_*CT*_ = − 0.041	0.337	B.R.	11.10	*Φ*_*CT*_ = 0.111	0.108	B.R.	− 514	*Φ*_*CT*_ = − 0.051	0.489
Archipelago	B.P.	14.47	*Φ*_*ST*_ = 0.169	< 0.001	B.P.	16.72	*Φ*_*ST*_ = 0.127	< 0.001	B.P.	3.74	*Φ*_*ST*_ = 0.148	0.008	B.P.	17.55	*Φ*_*ST*_ = 0.124	< 0.001
	W.P.	83.12	*Φ*_*SC*_ = 0.148	< 0.001	W.P.	87.34	*Φ*_*SC*_ = 0.161	< 0.001	W.P.	85.17	*Φ*_*SC*_ = 0.042	0.195	W.P.	87.59	*Φ*_*SC*_ = 0.167	< 0.001

*S.O.V.* source of variation, *B.R.* between regions, *B.P.* between populations within regions, *W.P.* within populations, % *var*. percentage of variation.

*n.b.* Tropical eastern pacific = Clipperton Island.

Pairwise AMOVAs of *E. diadema* mtDNA (Table 6) and nDNA (Table 7) revealed a ubiquitous pattern of genetic variation concentrated within populations. Greatest within-population variance in mtDNA occurred when these populations were compared between the tropical eastern Pacific and the Hawaiian Archipelago (94.68%, *Φ*_*SC*_ value = 0.103 *p* = < 0.001) and between the western Pacific Ocean with the Hawaiian Archipelago (94.84%, *Φ*_*SC*_ value = 0.073, *p* = 0.110). Comparisons between the Indian Ocean, the Hawaiian Archipelago and the western Pacific Ocean revealed greater interregional variance of nDNA than between populations, but fixation indices for nDNA were not significant (Table 7).

**Table 6:** Between region pairwise AMOVA of mtDNA Tamura and Nei^31^ distances in *Echinothrix diadema.*

	India	Ocean	Statistic	*p*	Central	Pacific	Ocean	*p*	Western	Pacific	Ocean	*p*	Tropical	eastern	Pacific	*p*
	S.O.V.	% var.			S.O.V.	% var.	Statistic		S.O.V.	% var.	Statistic		S.O.V.	% var.	Statistic	
Central	B.R.	− 0.33	*Φ*_*CT*_ = − 0.003	0.423												
Pacific	B.P.	22.89	*Φ*_*ST*_ = 0.226	< 0.001												
Ocean	W.P.	77.44	*Φ*_*SC*_ = 0.228	< 0.001												
Western	B.R.	− 3.42	*Φ*_*CT*_ = − 0.034	0.736	B.R.	− 7.65	*Φ*_*CT*_ = − 0.076	0.934								
Pacific	B.P.	16.42	*Φ*_*ST*_ = 0.130	< 0.001	B.P.	27.38	*Φ*_*ST*_ = 0.197	< 0.001								
Ocean	W.P.	87.00	*Φ*_*SC*_ = 0.159	< 0.001	W.P.	80.28	*Φ*_*SC*_ = 0.254	< 0.001								
Tropical	B.R.	2.86	*Φ*_*CT*_ = 0.029	0.274	B.R.	− 4.21	*Φ*_*CT*_ = − 0.042	0.666	B.R.	− 0.65	*Φ*_*CT*_ = − 0.006	1.000				
Eastern	B.P.	16.98	*Φ*_*ST*_ = 0.198	< 0.001	B.P.	26.78	*Φ*_*ST*_ = 0.226	< 0.001	B.P.	7.60	*Φ*_*ST*_ = 0.069	0.004				
Pacific	W.P.	80.16	*Φ*_*SC*_ = 0.175	< 0.001	W.P.	77.43	*Φ*_*SC*_ = 0.257	< 0.001	W.P.	93.04	*Φ*_*SC*_ = 0.076	0.016				
Hawaiian	B.R.	− 2.38	*Φ*_*CT*_ = − 0.024	0.395	B.R.	− 12.37	*Φ*_*CT*_ = − 0.124	0.830	B.R.	− 2.10	*Φ*_*CT*_ = − 0.021	0.650	B.R.	− 5.32	*Φ*_*CT*_ = − 0.053	1.000
Archipelago	B.P.	14.98	*Φ*_*ST*_ = 0.126	< 0.001	B.P.	28.84	*Φ*_*ST*_ = 0.165	< 0.001	B.P.	7.42	*Φ*_*ST*_ = 0.053	0.018	B.P.	10.81	*Φ*_*ST*_ = 0.055	< 0.001
	W.P.	87.40	*Φ*_*SC*_ = 0.146	< 0.001	W.P.	83.53	*Φ*_*SC*_ = 0.257	< 0.001	W.P.	94.68	*Φ*_*SC*_ = 0.073	0.110	W.P.	94.52	*Φ*_*SC*_ = 0.103	< 0.001

*S.O.V.* source of variation, *B.R.* between regions, *B.P.* between populations within regions, *W.P.* within populations, % *var*. percentage of variation.

*n.b.* Tropical eastern pacific = Clipperton Island + Isla del Coco.

**Table 7:** Between region pairwise AMOVA of nDNA Tamura and Nei^31^ distances in *Echinothrix diadema.*

	India	Ocean	Statistic	*p*	Central	Pacific	Ocean	*p*	Western	Pacific	Ocean	*p*	Tropical	eastern	Pacific	*p*
	S.O.V.	% var.			S.O.V.	% var.	Statistic		S.O.V.	% var.	Statistic		S.O.V.	% var.	Statistic	
Central	B.R.	− 1.15	*Φ*_*CT*_ = −0.011	0.703												
Pacific	B.P.	− 1.01	*Φ*_*ST*_ = − 0.021	0.616												
Ocean	W.P.	102.16	*Φ*_*SC*_ = − 0.009	0.520												
Western	B.R.	1.89	*Φ*_*CT*_ = 0.019	0.205	B.R.	− 4.02	*Φ*_*CT*_ = − 0.040	1.000								
Pacific	B.P.	− 3.94	*Φ*_*ST*_ = − 0.020	0.697	B.P.	− 2.28	*Φ*_*ST*_ = − 0.063	0.734								
Ocean	W.P.	102.05	*Φ*_*SC*_ = − 0.040	0.679	W.P.	106.30	*Φ*_*SC*_ = 0.022	0.603								
Tropical	B.R.	− 3.08	*Φ*_*CT*_ = − 0.031	0.936	B.R.	1.22	*Φ*_*CT*_ = 0.012	0.413	B.R.	− 1.50	*Φ*_*CT*_ = − 0.015	1.000				
Eastern	B.P.	0.39	*Φ*_*ST*_ = − 0.027	0.558	B.P.	1.16	*Φ*_*ST*_ = 0.024	0.266	B.P.	4.25	*Φ*_*ST*_ = 0.027	0.221				
Pacific	W.P.	102.69	*Φ*_*SC*_ = 0.004	0.397	W.P.	97.62	*Φ*_*SC*_ = 0.012	0.218	W.P.	97.26	*Φ*_*SC*_ = 0.042	0.149				
Hawaiian	B.R.	3.07	*Φ*_*CT*_ = 0.031	0.413	B.R.	− 1.75	*Φ*_*CT*_ = − 0.017	0.495	B.R.	4.41	*Φ*_*CT*_ = 0.044	0.327	B.R.	− 12.56	*Φ*_*CT*_ = − 0.125	1.000
Archipelago	B.P.	− 0.05	*Φ*_*ST*_ = 0.030	0.219	B.P.	− 0.22	*Φ*_*ST*_ = − 0.019	0.527	B.P.	− 12.36	*Φ*_*ST*_ = − 0.079	0.985	B.P.	18.86	*Φ*_*ST*_ = 0.063	0.052
	W.P.	96.98	*Φ*_*SC*_ = − 0.001	0.631	W.P.	101.97	*Φ*_*SC*_ = 0.002	0.472	W.P.	107.96	*Φ*_*SC*_ = − 0.129	1.000	W.P.	93.70	*Φ*_*SC*_ = 0.167	0.098

*S.O.V.* source of variation, *B.R.* between regions, *B.P.* between populations within regions, *W.P.* within populations, % *var *. percentage of variation.

*n.b.* Tropical eastern pacific = Clipperton Island + Isla del Coco.

### Isolation by geographic distance

A significant correlation between pairwise *Φ*_*ST*_ and geographic distance was found in the mtDNA of *Echinothrix calamaris* clade 1 (*r* = 0.277, *p* = < 0.042) but not in the nDNA (*r* = − 0.033, *p* < 0.820). No significant correlation was found in either clade 3 of *E. calamaris* (Pacific) (mtDNA *r* = − 0.014, *p* = 0.960; nDNA *r* = 0.051, *p* = 0.868) or *E. diadema* (mtDNA *r* = − 0.238, *p* = 0.286; nDNA *r* = − 0.047, *p* = 0.877). A Mantel test was not employed to correlate pairwise *Φ*_*ST*_ and geographic distance for clade 2 of *E. calamaris* as only two populations were sampled in this study.

### Colour polymorphism

Colour variation was extensive among *E. calamaris* specimens, but not among those of *E. diadema* (Fig. 1). Juvenile *E. diadema* had green and purple-banded interambulacral and ambulacral spines, the test epithelial tissues were burgundy or black (lighter at night) with a black periproctal cone. In adults the colour of the test epithelial tissues was black, the interambulacral spines were black or banded dark purple and green, but always with a blue, green, or turquoise iridescent sheen. The ambulacral spines were banded in all *E. diadema*, regardless of age.

**Figure 6:**
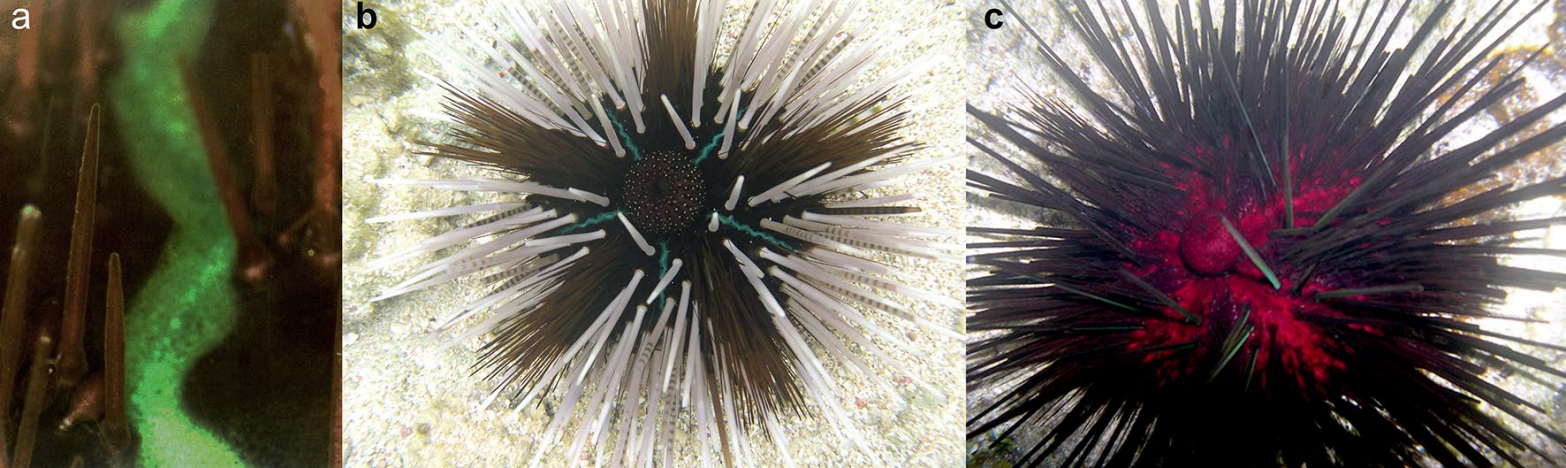
Green median regions of interambulacra in *E. calamaris*. (**a**) Close-up of median region, (**b**) white colour morph with green median regions (haplotype = clade 1, Namatakula, Fiji 13), (c) red colour morph with only red pigmentation in epithelial tissues of interambulacra (haplotype = clade 1, Namatakula, Fiji 22).

In clades 1 and 3 of *E. calamaris* colour variation within each clade included members that were white, green, red, pink, purple, brown or black, with a variety of combinations, but was not clade or locality specific. Colour variation in clade 2 of *E. calamaris* was reduced. Individuals from Dahab in the Red Sea had predominantly black interambulacral spines and test epithelial tissues that were either red or black. Those from the Gulf of Oman were black with a red tinge typical of the black colour morphs reported from the Gulf of Aqaba by Dafni^32^. In contrast to *E. diadema,* the ambulacral spines of *E. calamaris* were typically one colour with a dark venom gland at the tip, but not banded in either adults or juveniles. Bright green median regions of the interambulacra (Fig. 6a, b), present only in *E. calamaris,* may advertise the venomous nature of the ambulacral spines^27,28^. However, these median regions were absent in some red and black colour morphs (Fig. 6c), as were white/yellow spots on the skin of the periproctal cone.

At Sosoikula Reef, Suva, Fiji, five *E. calamaris* were members of clade 1, nineteen of clade 3, while a single specimen was a hybrid of *E. diadema* (nDNA) and *E. calamaris* mtDNA clade 3 (Fig. 1). The hybrid’s periproctal cone was black and small, resembling that of *E. diadema*. The spines around the edge of the interambulacra and down the entire ambulacra were banded black and grey, with a distinct blue/green sheen similar to *E. diadema*. All other interambulacral spines were white with no banding. Spine structure, with verticillations on the interambulacral spines were typical of *E. calamaris *sensu Coppard and Campbell^28^, and two forms of tridentate pedicellariae that were structurally closest to *E. calamaris* (see Coppard and Campbell^33^).

## Discussion

Similar to other diadematid echinoids, *Echinothrix* has a poor fossil record primarily due to the fragile nature of the test that breaks-up and disperses after death^29^. A paucity of fossil material has previously made it hard to determine when species diverged. Our analysis, using combined dating from fossil spines and substitution rates from the closely related genus *Diadema* Gray, 1825^34^ estimated that the ancestors of *E. diadema* and *E. calamaris* diverged ~ 16.8 Ma in the Burdigalian stage of the early Miocene. This was a period of major climatic and oceanographic change^35^, with global sea level fluctuations due to the onset of the middle Miocene cooling and the East Antarctic ice sheet expansion^36,37^. These events coincided with the closure of the Tethys seaway 20–13.8 Ma, which resulted in oceanographic changes and the reorganization of oceanographic currents^38^. Collision of the Australia and New Guinea plate with the Asian plate also changed ocean circulation patterns in the Indo-Malayan region and significantly altered genetic connections among marine populations^39,40^. We propose that the post-collision exposure of the Sunda Shelf and low sea level stands in the late Miocene resulted in the split between Indian Ocean and Pacific Ocean *E*. *calamaris* ~ 9.8 Ma, with a later range expansion into the Pacific by the Indian Ocean + Pacific Ocean clade.

The Red Sea and Gulf of Oman clade 2 of *Echinothrix calamaris* diverged from those in the Indian Ocean ~ 7.2 Ma in the late Tortonian stage of the Miocene. The confamiliar *Diadema setosum* (Leske, 1778) also has a clade endemic to the shores of the Arabian Peninsula that was estimated as having diverged from the Indo-Pacific clade 2.5–7.2 Ma^34^. Following probable isolation and divergence in the Red Sea, clade 2 later spread along the Arabian Peninsula into the Gulf of Oman and possibly into the much younger Persian Gulf (not sampled in this study), when the Strait of Hormuz opened 14,000 years ago^41^. Habitat suitability both within the Red Sea and along the South of the Arabian Peninsula was likely to have varied during glacial cycles and resulted in population contractions and expansions. Less hospitable conditions of increased salinity and water temperatures in the Red Sea from the Miocene through the Pleistocene were survived by many marine lineages^42^, and *E. calamaris* may have been one of them. Alternatively, *E. calamaris* may have re-established itself in the Red Sea from populations around the Arabian Peninsula and Gulf of Oman when conditions became more favourable.

Both clade 1 of *E. calamaris* and *E. diadema* have extensive distributions throughout the Indo-Pacific, while clades 2 and 3 of *E. calamaris* are restricted to specific geographic regions. A trend of isolation by distance was observed only in clade 1 of *E. calamaris*, but not in *E. diadema*, which remains genetically connected across ~ 26,000 km of the Indo-Pacific*.*

Larval duration of *Echinothrix* in the plankton is unknown. Time to metamorphosis in other diadematids appears to vary depending on water temperature. Larvae of tropical *Diadema antillarum* Philippi, 1845 settle in the laboratory in ~ 36 days^43^, but the larvae of temperate *Centrostephanus rodgersii* (A. Agassiz, 1864) spend between 105 and 126 days in the water column before settlement^44^. If water temperature is a factor in development rate, then we should expect members of *Echinothrix* to be closer to *Diadema* than *Centrostephanus* Peters, 1855. However, time to metamorphosis may vary among clades of *Echinothrix* and among geographic regions.

In the Indian Ocean, a high degree of genetic isolation (based on mtDNA) was exhibited by the population of *E. calamaris* at Réunion Island, with genetic connections only with that at Madagascar, corresponding to the Madagascar ecoregion of Spalding et al*.*^45^. High population connectivity was found between Indian Ocean and western Pacific populations of *E. diadema*, but gene flow was somewhat restricted in clade 1 of *E. calamaris*. Today, the Strait of Malacca seaway through the Malay-Indonesian Archipelago has sufficient current activity to allow gene flow among some Indo-Pacific marine species^46^. The Indonesian throughflow between Malaysia and Australia also has the potential to distribute plankton westwards^47^ and allow connectivity between Indian Ocean and Pacific Ocean *Echinothrix*.

Clade 3 of *E. calamaris* had a more restricted distribution than clade 1 or *E. diadema*, from Malaysia in the West to Polynesia in the East (not present in Hawaii, at Kingman Reef or in the tropical eastern Pacific), with restricted connectivity between western and central Pacific populations. Despite having a large population on the Pacific coast of Malaysia and a population in the Coral Sea off Papua New Guinea, clade 3 of *E. calamaris* has not entered the Indian Ocean. The ability of clade 3 *E. calamaris* larvae to disperse either North of Papua New Guinea or through the Torres Strait between Northern Australia and Papua New Guinea is unknown. However, the complex eddies around reefs and islands and the seasonal reversal of currents^48,49^ may impart stochasticity of larval movement into the Indian Ocean.

Differences in the distributions of clade 1 and clade 3 could not be explained by their age, as they are sister clades. Members of the two clades may have different ecological requirements. Alternatively, different distributions may be the result of chance dispersal events. Crossing the uninterrupted deep water that forms the Eastern Pacific Barrier (EPB) (Fig. 2) is very difficult for shallow water marine invertebrates^18–21^. In this study, we found that both *E. diadema* and clade 1 of *E. calamaris* have crossed the EPB. Shortened transport time across the EPB resulting from a strong El Niño event is a likely mechanism for dispersal^17,21,50–56^. We propose that the very strong 1997–1998 El Niño event may well have facilitated dispersal of *Echinothrix* larvae across the EPB and corresponds to the last colonization by *Echinothrix* larvae in the eastern Pacific.

Gene flow between Clipperton Island and Isla del Coco was not quantified in *E. calamaris* due to the limited sample size from Isla del Coco. However, in *E. diadema* gene flow appeared to be restricted (Table S3). Crossing the eastward flowing North Equatorial Counter Current (NECC) would severely limit exchange between Isla del Coco and Clipperton but potentially facilitate some exchange between Isla del Coco and central and South America. *Echinothrix* has recently been reported from Colombia including Malpelo Island and Gorgona Island^57^, but to date no *Echinothrix* have been recorded from Panama^58^.

Haplotype networks and AMOVA indicated the presence of similar haplotypes at Clipperton Island and the Hawaiian Archipelago in clade 1 of *E. calamaris*. This is most likely explained by the survival of larvae carried by the North Equatorial Current (NEC) from Clipperton Island towards the southeast of the Hawaiian Archipelago^59^. In *E. diadema* no restriction to gene flow was found between Clipperton Island and the Hawaiian Archipelago (Table S3), perhaps because larvae of *E. diadema* have greater dispersal ability than those of *E. calamaris*.

The Line Islands, including eight islands that form part of Kiribati, lie 1800 km south of Hawaii and have been proposed as a source for larval dispersal to the Hawaiian Archipelago^60^. In non-El Niño years equatorial upwelling north of Kiribati^61,62^ in conjunction with the westward-flowing South Equatorial Current and the eastward-flowing North Equatorial Counter Current limit larval movement north and northeast. Lack of recent gene flow in *E. calamaris* between populations at Kiribati with populations in the tropical eastern Pacific and at Kingman Reef was apparent, both through large and significant *Φ*_*ST*_ values and limited connectivity in the mtDNA haplotype network. This pattern was also found in *E. diadema* (Table S4). However, populations of both *E. calamaris* and *E. diadema* at Kingman Reef showed evidence of gene flow with both the Island of Hawaii and Clipperton Island, and therefore Kingman Reef may act as a larval source for dispersal north and east.

Evidence of gene flow between Kiribati and Moorea Island to the southeast was found in both clade 1 and clade 3 of *E. calamaris*. Kiribati is influenced by the eastward-flowing Equatorial Counter Current (ECC) and the westward-flowing South Equatorial Current (SEC)^63,64^. These currents are reported to have transported to Kiribati floating volcanic pumice and presumably larvae derived from Krakatau (western Pacific), the Tonga Trench (southwestern Pacific) and Benedicto Island, Mexico (eastern Pacific)^65^.

Gene flow in mtDNA between Japan and Kiribati and between Japan and Moorea in both clades 1 and 3 of *E. calamaris* is hard to explain by the flow of oceanic currents. This is also true for gene flow in *E. diadema* between Taiwan and both the tropical eastern Pacific and the Hawaiian Archipelago, and between the Seychelles and Isla del Coco.

Clade 2 of *E. calamaris* was endemic to the Red Sea and Gulf of Oman. *Echinothrix diadema* was not encountered at either location in the limited collections of this study. Mastaller’s^66^ report of *E. diadema* in the Gulf of Aqaba is thought to be an error resulting from misidentification of the dark morph of *E. calamaris* as *E. diadema*^32^. No mtDNA haplotypes of clade 2 were found among *E. calamaris* in either the Indian or Pacific Ocean populations. Large and significant *Φ*_*ST*_ values indicated that Red Sea and Gulf of Oman populations were isolated from one another. Today larval dispersal of the Red Sea population of *E. calamaris* is probably restricted by upwelling of cold, nutrient-rich water between the Gulf of Aden and the Arabian Sea, which acts as a significant barrier to gene flow in other species^42^. Upwelling off Oman in conjunction with seasonal current patterns result in large fluctuations in temperature and primary productivity^67–69^ and may limit gene flow between clade 2 of *E. calamaris* in the Gulf of Oman with other populations.

Discordance in population structure between mtDNA and nDNA cannot completely be explained by different evolutionary rates between markers. Although nuclear 28S rDNA appeared to be evolving at a slow rate (Table 1), the nDNA Calpain-7 intron was found to be evolving at a similar rate to mitochondrial 16S, with only slightly smaller genetic distances between clades (Table 1). This raises the question of whether differences in genetic structure between mtDNA and nDNA have resulted from sex-biased migration of their pelagic larvae. In such a scenario, male larvae would remain longer in the plankton resulting in increased connectivity and gene flow in biparentally inherited nDNA relative to matrilineally inherited mtDNA. To-date sexual dimorphism in echinoid larvae has not been demonstrated and the question of sex-biased migration remains open.

In disagreement with the literature^13,22–24^, variation in colouration of *E. diadema* was slight throughout its geographic range. This was in stark contrast to *E. calamaris,* which exhibited diverse colour variation within clades 1 and 3, with no indication that colour polymorphisms were correlated with either clade or geographic location. Why two such ecologically similar species displayed such different levels of colour variation is puzzling. *Echinothrix diadema* and *E. calamaris* occur in mixed species aggregations under coral heads or in large crevices^14^ and have very similar dietary preferences^15^. It is therefore unlikely that pigments from food affect their colouration. Both species reportedly grow to a large size, with recorded horizontal test diameters of 143 mm in *E. calamaris* and 110 mm in *E. diadema*^70^. Both species have venomous ambulacral spines to ward off predators^28^. There is no apparent explanation for the diversity of colour seen in contemporary *E. calamaris*; it is impossible to know whether it may be a remnant of past evolutionary processes that are no longer present.

Our molecular phylogeny revealed the presence of three distinct and genetically distant clades in *Echinothrix calamaris,* that suggested species level differentiation. Although not a direct comparison, genetic distances in ATPase-6 and ATPase-8 between clades of *E. calamaris* (Table 1) were greater than those reported in the same genes between some species of *Diadema* (e.g. 4.34% between *D. savignyi* (Audouin, 1809) and *D. mexicanum* A. Agassiz, 1863; 2.61% between *D. savignyi* and *D. paucispinum-a* A. Agassiz, 1863)^34^. Time since divergence between clades of *E. calamaris* (~ 9.8 million years (My) between clade 1 + 2 and clade 3; ~ 7.2 My between clade 1 and clade 2) was also greater than between these species of *Diadema* (less than 4 My). However, we have no information on reproductive isolation between clades. Clade 1 and clade 3 have overlapping distributions in parts of the western and central Pacific (Fig. 2) and are living side-by-side on coral reefs with no spatial isolation, so their genetic divergence may well be the result of reproductive incompatibility. Slight differences in tridentate pedicellarial morphology have been reported among members of *E. calamaris*^33^, but we could find no major morphological differences that would be diagnostic between clades. In Fiji, where both clades 1 and 3 occur, *E. calamaris* spawned around the time of the new moon^71^. Only two individuals were found by Coppard and Campbell^71^ to have spawned out of phase with conspecifics on the full moon. Given the lack of temporal or spatial isolation between clades, possible barriers to genetic intermingling could be molecules involved in species-specific recognition of gametes or lower hybrid fitness.

We have found hybrids between *E. diadema* and *E. calamaris* to be rare, quite possibly because the two species spawn fifteen days out of phase with each other^71^. A single *E. diadema*/*E. calamaris* clade 3 hybrid was found at Suva, Fiji. Very occasional asynchrony among *E. calamaris* spawning out of phase with conspecifics on the full moon simultaneously with *E. diadema* may partially explain such introgression. It is likely that close proximity of spawning individuals would be necessary, and that other reproductive barriers such as those generated by proteins involved in sperm and egg attraction^72^ and sperm and egg binding and fusion including sperm bindin^73^ and its egg receptor EBR1^74^ would have to be overcome for this to occur.

## Methods

**Figure 7:**
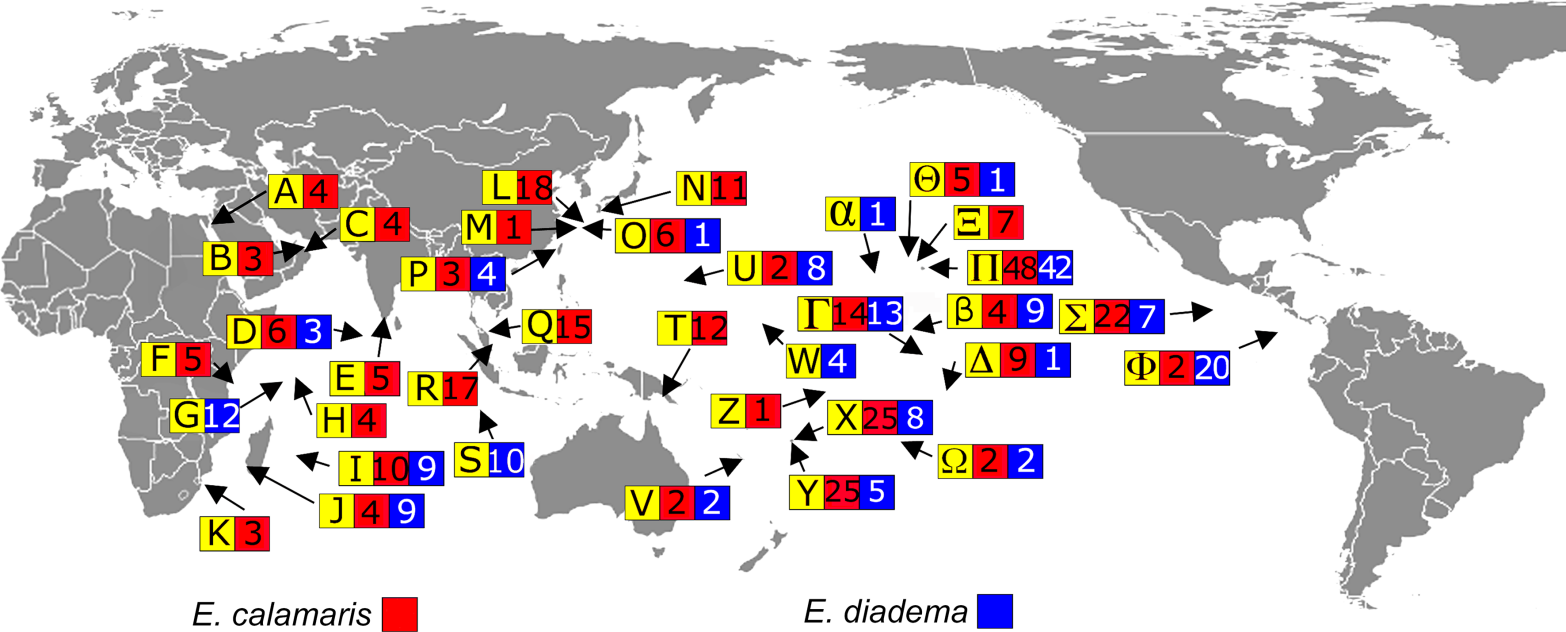
Collection localities of *Echinothrix* used in this study. Letter refers to locality (see Fig. 2 legend), number to the sample size, and colour to species. The outline of this map was downloaded from Dmthoth, CC BY-SA 3.0, via Wikimedia Commons and edited in Photoshop Elements 2020.

Two hundred and ninety-nine *Echinothrix calamaris* and 171 *E. diadema* were collected from localities throughout the Indo-Pacific (Fig. 7). Samples represented the diversity of colour morphs present at each location. Gonads and muscle from the Aristotle’s lantern were dissected and preserved in either 95% ethyl alcohol or high salt dimethyl sulfoxide buffer. *Astropyga pulvinata* was also sampled from off Uva Island, Gulf of Chiriquí Panama (7.8016° N, 81.7579° W) for use as an outgroup. Collection information including tissue voucher numbers at the Smithsonian Tropical Research Institute, Panama are included in Supplementary Table S8.

### DNA extraction, sequencing, and alignment

Genomic DNA was extracted from the lantern muscle or gonad using a DNeasy tissue kit (Qiagen) or proteinase K microextractions. Mitochondrial 16S rDNA, ATPase-6, and ATPase-8, as well as nuclear 28S rDNA and the intron in Calpain-7 were amplified for all samples. 16S rDNA was amplified using the 16Sar and 16Sbr primers of Kessing et al.^75^ and the ATPase-6 and ATPase-8 regions using the LYSa (Lysine-tRNA) and ATP6b primers of Lessios et al*.*^34^. Both mitochondrial regions were amplified in PCR reactions containing 0.2–0.5 µl of extracted genomic DNA (approximately 10–15 ng), 12.0 µl of nuclease free H_2_0 (adjusted to 12.3 µl when using less genomic DNA), 5.0 µl GoTaq^®^ Flexi Buffer (5 ×), 2.5 µl MgCl_2_ (25 mM), 2.5 µl dNTPs (8 mM), 1.25 µl (10 mM) of each of the forward and reverse primer, and 0.6 units of Flexi-GoTaq^®^ polymerase (Promega)^®^. The mixture was cycled 39 times at 96 °C for 10 s, 94 °C for 30 s, 50 °C for 45 s, 72 °C for 1 min, with a final extension at 72 °C for 5 min.

Nuclear 28S rDNA was amplified using the primers and protocol of Littlewood and Smith^76^. Calpain-7 intron primers were designed for *Echinothrix* based on the i21 EPIC (Exon Priming Intron Crossing) primers of Gerard et al.^77^. The intron was amplified using the forward primer 5′-CCGGTATACAATCCTTGTGGC and reverse primer 5′-AGCGACACCCAGAATTCGTT. The PCR reaction mixture and conditions were the same as used for mtDNA, but with the annealing temperature raised to 55 °C. PCR products of 28S and the Calpain-7 intron were cloned using Promega pGEM-T Easy kits to avoid polymorphisms. One clone was amplified from each specimen using Promega M13 and M13R primers. After purification in Sephadex columns, amplification with the same primers, and labelling with Applied Biosystems (ABI) Prism BigDye terminators, nucleotides were sequenced in both directions in an ABI 3130 XL automatic sequencer. Alignments were performed in MacClade^78^.

After trimming sequence ends of ambiguous bases and overlapping segments, there were 614 bp of 16S rDNA, 174 bp of ATPase-8, 360 bp of ATPase-6, 1180 bp of 28S rDNA and 277 bp of the Calpain-7 intron, including protein binding site and 84 bp of the flanking 3’ exon. The first ~ 200 bases from the 5’ end of the Calpain-7 intron were deleted from the original 477 bp due to ambiguous low-quality sequence reads. This resulted in 2605 bp of DNA sequence for each specimen.

### Phylogenetic analyses

Unique haplotypes were identified in MacClade^79^, and redundant sequences were removed. jModeltest v. 2.1.1^80^ was employed to determine the best model of molecular evolution for each DNA region based on the AIC criterion^81^. Models selected were 16S: HKY + I + G (I = 0.4530, *α* = 0.8020), ATPase-8: TIM3 + I (I = 0.4060), ATPase-6: GTR + I + G (I = 0.2120, *α* = 0.5020), 28S: TIM2 + I (I = 0.9540) and Calpain-7 intron: GTR + G (*α* = 0.3560). The data were concatenated, and a partitioned Bayesian phylogenetic analysis was carried out with MrBayes v. 3.2^82^ using for each gene region the model suggested by jModeltest and parameters unlinked across partitions. A single haplotype of *Astropyga pulvinata* was chosen as an outgroup. The analysis was started with Dirichlet priors for rates and nucleotide frequencies and run for 6 × 10^8^ steps, sampling every 3000th tree from two runs. Convergence was assessed according to the average standard deviation of split frequencies reaching < 0.01 and potential scale reduction factor^83^ 1.00 for all parameters. The runs were also visually checked for a lack of trends in Tracer v1.6^78^. The first 25% of trees were discarded from each run as burn-in, and a 50% majority rule tree was constructed. Maximum likelihood (ML) analysis was conducted on the concatenated data in RAxML v.8.2.x^84^ using the GTR + G model and rapid bootstrapping for 10,000 iterations. Nodes with less than 85% Bayesian support in MrBayes and less than 75% ML support in RAxML were collapsed.

Haplotype networks are a means of visualizing relationships when the ancestors of extant groups are assumed to be still present. Median joining haplotype networks for each major clade, combining mtDNA genes in one analysis (Fig. 4) and combining nDNA sequences in a separate analysis (Fig. 5), were constructed in PopART (Population Analysis with Reticulate Trees)^85^, with reticulation tolerance set to zero. The mean genetic distance between clades according to the models suggested by jModeltest was calculated from all pairwise comparisons estimated by Paup*^86^.

### Timing of divergence

A partitioned analysis of unique haplotypes of ATPase-6 (360 bp) and ATPase-8 (174 bp) from 237 taxa was analysed in BEAST (v1.10.4.)^87^. Substitution models for the reduced datum set selected by jMODELTEST were GTR + I for ATPase-6 and TN93 + I for ATPase-8. BEAST was run using an uncorrelated relaxed clock with a lognormal distribution and a Yule speciation process. A single haplotype of *Astropyga pulvinata* was chosen as an outgroup. Substitution rates (per site, per million years) for ATPase-6 and ATPase-8 from the closely related genus *Diadema* were used to calibrate the phylogeny, based on the assumption that the western Atlantic *D. antillarum* was separated from the eastern Pacific *D. mexicanum* 2.5 Ma^34^. Initial ucldmean clock rates were set to 0.0176 substitutions per site per million years for ATPase-6 and 0.0108 substitutions per site, per million years for ATPase-8. An exponential prior with an offset of 15.97 and a mean of 2.0 was used to constrain the minimum age of *Echinothrix*. This was based on the 15.7 to ~ 25 Ma Burdigalian, early Miocene age of the *Echinothrix* spine fragment from Java, Indonesia^29^. The results from five individual runs, each of 10–50 × 10^6^ steps, logged every thousand steps were combined in LogCombiner v. 1.10.4 with 10% burnin from each run, resulting in 120,000 trees. The runs were visually checked for lack of trends in Tracer v.1.6^79^. The effective sample size (ESS) of the combined set was > 212 for all parameters except ATP6ucld.stdv, which had a value of 129.

### Gene flow between populations and biogeographic regions

*Φ*_*ST*_ statistics represent divergence between populations relative to divergence within populations and are assumed to measure degree of on-going gene flow. We estimated gene flow separately from concatenated mitochondrial and concatenated nuclear genes, because of the four-fold difference in effective population size. Pairwise *Φ*_*ST*_ values using Tamura and Nei^31^ distances were calculated in Arlequin v. 3.5.2.2^88^ between geographic populations within clades. Analysis of molecular variance (AMOVA) using Tamura and Nei^31^ distances was estimated in Arlequin from 1023 random reshufflings of haplotypes in each clade. *Φ*-statistics were then employed to estimate the proportion of genetic variability found between geographic regions (*Φ*_*CT*_), between populations within geographic regions (*Φ*_*ST*_) and within populations (*Φ*_*SC*_). Geographic regions were defined as the Indian Ocean, western Pacific Ocean (Malay-Indonesian Archipelago to Guam) central Pacific Ocean (New Caledonia, East to Moorea), Hawaiian Archipelago and the tropical eastern Pacific Ocean (Isla del Coco and Clipperton Island). Geographic sea distances between collection points were measured using Google Maps. Standard Mantel^89^ tests were conducted to evaluate possible relationships between geographic distance and genetic divergence with significance tested using 10,000 permutations.

### Equipment and settings

Images of sea urchins in Figs. 1 and 6 were taken by S.E.C., M. Barton, O. Bronstein, and F. Ducarme. Brightness, colour, and contrast were unchanged.

## Supplementary Information

Supplementary Information 1.

Supplementary Information 2.

## Data Availability

The data generated and or analysed during the current study are available in GenBank with the accession numbers MW324008-MW324478 for 16S, MW329044-MW329514 ATPase-8, MW329515-MW329985 ATPase-6, MW324711-MW325181 28S and MW410240-MW410710 for Calpain-7 intron. The alignment of the concatenated haplotypes of all genes is available from the Dryad repository at https://doi.org/10.5061/dryad.v15dv41wg.
