# Supplementary material for: Phylogeography, colouration, and cryptic speciation across the Indo-Pacific in the sea urchin genus Echinothrix
Source: Sci Rep. 2021 Aug 16;11:16568. doi: 10.1038/s41598-021-95872-0 (PMC8368070; doi:10.1038/s41598-021-95872-0)
Supplement: Supplementary file 1 — Supplementary Information 1. [file 41598_2021_95872_MOESM1_ESM.docx]

Supplementary Tables S1 to S7. Population pairwise *Φ_ST_* values based on Tamura & Nei^34^ distances for the concatenated ATPase6, ATPase8 and 16S mtDNA and concatenated 28S and Calpain-7 Intron nuclear DNA sequences.

Table S1. *Echinothrix calamaris* clade 1 (Indo-Pacific) mtDNA *= *p* <0.05 and **= *p* <0.01

|  | Maui,  Hawaii | Oahu,  Hawaii | Hawaii,  Hawaii | Clipper.  Island | Kingman Reef | Malay. | Japan | Moorea  Island | Kiribati | Suva,  Fiji | Namat.,  Fiji | Zanzibar | Seych. | India | Maldives | Madagas. | Réunion  Island |
| --- | --- | --- | --- | --- | --- | --- | --- | --- | --- | --- | --- | --- | --- | --- | --- | --- | --- |
| Oahu, Hawaii | 0.444** |  |  |  |  |  |  |  |  |  |  |  |  |  |  |  |  |
| Hawaii, Hawaii | 0.014 | 0.286** |  |  |  |  |  |  |  |  |  |  |  |  |  |  |  |
| Clipperton Island | 0.069* | 0.361** | 0.100** |  |  |  |  |  |  |  |  |  |  |  |  |  |  |
| Kingman Reef | 0.105* | 0.497** | -0.014 | 0.065 |  |  |  |  |  |  |  |  |  |  |  |  |  |
| Malaysia | 0.679** | 0.786** | 0.444** | 0.520** | 0.697* |  |  |  |  |  |  |  |  |  |  |  |  |
| Japan | 0.585** | 0.657** | 0.490** | 0.529** | 0.569* | 0.039 |  |  |  |  |  |  |  |  |  |  |  |
| Moorea Island | 0.682** | 0.777** | 0.492** | 0.551** | 0.709* | 0.231* | 0.096 |  |  |  |  |  |  |  |  |  |  |
| Kiribati | 0.613** | 0.704** | 0.475** | 0.525** | 0.613* | 0.014* | 0.007 | 0.040 |  |  |  |  |  |  |  |  |  |
| Suva_Fiji | 0.628** | 0.697** | 0.479** | 0.542** | 0.574** | 0.046 | 0.173** | 0.204* | 0.157** |  |  |  |  |  |  |  |  |
| Namatakula Fiji | 0.648** | 0.717** | 0.503** | 0.566** | 0.613** | 0.040 | 0.225** | 0.301** | 0.223 ** | 0.058 |  |  |  |  |  |  |  |
| Zanzibar | 0.775** | 0.830** | 0.603* | 0.659** | 0.797* | 0.631* | 0.458** | 0.670** | 0.521** | 0.543** | 0.549** |  |  |  |  |  |  |
| Seychelles | 0.624** | 0.664** | 0.550** | 0.589** | 0.581* | 0.228 | 0.188* | 0.310* | 0.221* | 0.236* | 0.357** | 0.020 |  |  |  |  |  |
| India | 0.747** | 0.802** | 0.588** | 0.645** | 0.761** | 0.639** | 0.491** | 0.688** | 0.563** | 0.583** | 0.580** | 0.139 | 0.129 |  |  |  |  |
| Maldives | 0.540** | 0.612** | 0.492** | 0.531** | 0.518** | 0.1445 | 0.126 | 0.283** | 0.173* | 0.299** | 0.329** | 0.217* | 0.108 | 0.146 |  |  |  |
| Madagascar | 0.694** | 0.741** | 0.537** | 0.590** | 0.703* | 0.408* | 0.148 | 0.412* | 0.244* | 0.359** | 0.366** | 0.609* | 0.277** | 0.601** | 0.256* |  |  |
| Réunion Island | 0.669** | 0.702** | 0.555** | 0.607** | 0.655** | 0.303** | 0.250** | 0.387** | 0.266** | 0.343** | 0.301** | 0.553** | 0.363** | 0.559** | 0.342** | -0.019 |  |
| South Africa | 0.669** | 0.729** | 0.548** | 0.593** | 0.653* | 0.292 | 0.115 | 0.412* | 0.214* | 0.329* | 0.361** | 0.295* | 0.035 | 0.246* | -0.029 | 0.293* | 0.288** |

Table S2. *Echinothrix calamaris* clade 2 (Red Sea, Gulf of Oman) *= *p* <0.05

| Red Sea | Gulf of Oman |
| --- | --- |
| mtDNA | 0.540* |
| nDNA | 0.861* |

Table S3. *Echinothrix calamaris* clade 3 (Pacific) mtDNA*= *p* <0.05 and **= *p* <0.01

|  | Tioman, Malaysia | Redang,  Malaysia | Sesoko,  Japan | Ishigaki. Japan | Kyushu, Japan | Namatakula,  Fiji | Suva,  Fiji | Papua New Guinea |  |
| --- | --- | --- | --- | --- | --- | --- | --- | --- | --- |
| Redang, Malaysia | -0.010 |  |  |  |  |  |  |  |  |
| Sesoko Japan | 0.091* | 0.177** |  |  |  |  |  |  |  |
| Ishigaki. Japan | -0.014 | 0.017 | 0.121* |  |  |  |  |  |  |
| Kyushu, Japan | 0.180 ** | 0.280** | 0.054 | 0.247* |  |  |  |  |  |
| Namatakula, Fiji | 0.358 ** | 0.434** | 0.354** | 0.369** | 0.448** |  |  |  |  |
| Suva, Fiji | 0.231 ** | 0.295** | 0.245** | 0.231** | 0.348** | 0.006 |  |  |  |
| Papua New Guinea | 0.281 ** | 0.335** | 0.350** | 0.195** | 0.458** | 0.278** | 0.205** |  |  |
| Kiribati | 0.101 * | 0.185** | 0.107* | 0.089 | 0.216** | 0.150* | 0.029 | 0.126* |  |
| Moorea Island | 0.050 | 0.173* | 0.003 | 0.119 | 0.048 | 0.333** | 0.198* | 0.325** | 0.029 |

Table S4. *Echinothrix diadema* mtDNA *= *p* <0.05 and **= *p* <0.01

|  | Hawaii,  Hawaii | Kingman Reef | Isla del  Coco | Clipperton  Island | Namatakula,  Fiji | Suva, Fiji | Réunion  Island | Madagascar | Kiribati | Keeling  Islands | Seychelles | Taiwan | Guam |
| --- | --- | --- | --- | --- | --- | --- | --- | --- | --- | --- | --- | --- | --- |
| Kingman Reef | -0.032 |  |  |  |  |  |  |  |  |  |  |  |  |
| Isla del Coco | 0.068** | 0.075* |  |  |  |  |  |  |  |  |  |  |  |
| Clipperton Island | 0.008 | 0.008 | 0.139** |  |  |  |  |  |  |  |  |  |  |
| Namatakula, Fiji | 0.162* | 0.189** | 0.321** | 0.257** |  |  |  |  |  |  |  |  |  |
| Suva_Fiji | 0.327** | 0.330** | 0.432** | 0.357** | 0.279 ** |  |  |  |  |  |  |  |  |
| Réunion Island | 0.236** | 0.224** | 0.367** | 0.239** | 0.249** | 0.321** |  |  |  |  |  |  |  |
| Madagascar | 0.107** | 0.077* | 0.196** | 0.197** | 0.379** | 0.414** | 0.299** |  |  |  |  |  |  |
| Kiribati | 0.148** | 0.134** | 0.149** | 0.187** | 0.301** | 0.399** | 0.356** | 0.205** |  |  |  |  |  |
| Keeling Islands | 0.078** | 0.027* | 0.164** | 0.073* | 0.188** | 0.270** | 0.222** | 0.085* | 0.176** |  |  |  |  |
| Seychelles | 0.056** | 0.019 | 0.094** | 0.125* | 0.319** | 0.383** | 0.290** | 0.038 | 0.130** | 0.042 |  |  |  |
| Taiwan | 0.008 | 0.019 | 0.132 | 0.027 | 0.339* | 0.326** | 0.328** | 0.280** | 0.167* | 0.041 | 0.140 |  |  |
| Guam | 0.073** | 0.028 | 0.173** | 0.103* | 0.134* | 0.274** | 0.150** | 0.091* | 0.199** | 0.055 | 0.055 | 0.105 |  |
| Marshall Islands | 0.139** | 0.071 | 0.227** | 0.189* | 0.359** | 0.389** | 0.320** | 0.191* | 0.193** | 0.110 | 0.179* | 0.222* | 0.117 |

Table S5. *Echinothrix calamaris* clade 1 (Indo-Pacific) nDNA *= *p* <0.05 and **= *p* <0.01

|  | Maui,  Hawaii | Oahu,  Hawaii | Hawaii,  Hawaii | Clipper.  Island | Kingman Reef | Malay. | Japan | Moorea  Island | Kiribati | Suva,  Fiji | Namat.,  Fiji | Zanzibar | Seych. | India | Maldives | Madagas. | Réunion  Island |
| --- | --- | --- | --- | --- | --- | --- | --- | --- | --- | --- | --- | --- | --- | --- | --- | --- | --- |
| Oahu, Hawaii | 0.023 |  |  |  |  |  |  |  |  |  |  |  |  |  |  |  |  |
| Hawaii, Hawaii | 0.080 | -0.064 |  |  |  |  |  |  |  |  |  |  |  |  |  |  |  |
| Clipperton Island | 0.020 | -0.024 | 0.025 |  |  |  |  |  |  |  |  |  |  |  |  |  |  |
| Kingman Reef | 0.222* | 0.028 | 0.135* | 0.069 |  |  |  |  |  |  |  |  |  |  |  |  |  |
| Malaysia | 0.230* | 0.109 | 0.106 | 0.137* | 0.052 |  |  |  |  |  |  |  |  |  |  |  |  |
| Japan | 0.208* | 0.075 | 0.162* | 0.096 | 0.046 | 0.036 |  |  |  |  |  |  |  |  |  |  |  |
| Moorea Island | 0.304** | 0.214 | 0.104 | 0.168* | 0.191* | 0.069 | 0.279* |  |  |  |  |  |  |  |  |  |  |
| Kiribati | 0.437** | 0.385** | 0.388** | 0.338** | 0.335* | 0.325** | 0.253** | 0.333 |  |  |  |  |  |  |  |  |  |
| Suva_Fiji | 0.161* | 0.045 | 0.144* | 0.062 | 0.005 | 0.061 | 0.001 | 0.187* | 0.235* |  |  |  |  |  |  |  |  |
| Namatakula Fiji | 0.069 | 0.025 | 0.055* | 0.041 | 0.146* | 0.140* | 0.178* | 0.120* | 0.383** | 0.082 |  |  |  |  |  |  |  |
| Zanzibar | 0.389** | 0.291* | 0.325** | 0.269** | 0.216* | 0.278* | 0.209* | 0.262 | -0.092 | 0.162 | 0.327** |  |  |  |  |  |  |
| Seychelles | 0.210* | -0.001 | 0.101 | 0.105 | 0.012 | -0.022 | 0.084 | 0.175 | 0.214 | -0.044 | 0.140** | 0.179* |  |  |  |  |  |
| India | 0.254** | 0.247* | 0.202* | 0.104 | 0.257* | 0.373* | 0.248* | 0.437* | 0.475* | 0.105 | 0.059 | 0.381* | 0.251 |  |  |  |  |
| Maldives | 0.143* | 0.167* | 0.209** | 0.085 | 0.211* | 0.232* | 0.111 | 0.318** | 0.384** | 0.123 | 0.070 | 0.343** | 0.185 | 0.007 |  |  |  |
| Madagascar | 0.178* | 0.001 | 0.059 | 0.028 | -0.112 | -0.063 | -0.075 | 0.074 | 0.130 | -0.095 | 0.086 | 0.001 | 0.104 | 0.152 | 0.124 |  |  |
| Réunion Island | 0.233** | 0.064 | 0.096** | 0.135** | 0.017 | -0.049 | 0.101 | 0.049 | 0.243* | 0.076 | 0.131** | 0.145 | 0.012 | 0.263* | 0.238** | -0.086 |  |
| South Africa | 0.198* | 0.247 | 0.179* | 0.048 | 0.183 | 0.370 | 0.239 | 0.510* | 0.507 | 0.071 | 0.001 | 0.372 | 0.265 | -0.168 | -0.057 | 0.104 | 0.242 |

Table S6. *Echinothrix calamaris* clade 3 (Pacific) nDNA *= *p* <0.05 and **= *p* <0.01

|  | Tioman, Malaysia | Redang,  Malaysia | Sesoko,  Japan | Ishigaki. Japan | Kyushu, Japan | Namatakula,  Fiji | Suva,  Fiji | Papua New Guinea | Kiribati |
| --- | --- | --- | --- | --- | --- | --- | --- | --- | --- |
| Redang, Malaysia | -0.005 |  |  |  |  |  |  |  |  |
| Sesoko Malaysia | -0.018 | 0.022 |  |  |  |  |  |  |  |
| Ishigaki. Japan | -0.020 | 0.061 | -0.040 |  |  |  |  |  |  |
| Kyushu, Japan | 0.016 | 0.017 | -0.025 | -0.014 |  |  |  |  |  |
| Namatakula, Fiji | 0.031 | 0.037 | 0.024 | 0.061 | 0.047 |  |  |  |  |
| Suva, Fiji | 0.007 | 0.033 | 0.027 | 0.013 | 0.043 | <0.000 |  |  |  |
| Papua New Guinea | 0.080* | 0.142** | 0.061 | -0.067 | 0.093* | 0.154** | 0.069* |  |  |
| Kiribati | 0.273 ** | 0.300 ** | 0.266** | 0.275** | 0.256** | 0.308** | 0.255** | 0.248** |  |
| Moorea Island | 0.004 | -0.015 | -0.028 | 0.013 | -0.025 | -0.096 | -0.036 | 0.111* | 0.249** |

Table S7. *Echinothrix diadema* nDNA *= *p* <0.05 and **= *p* <0.01

|  | Hawaii,  Hawaii | Kingman  Reef | Isla del  Coco | Clipperton  Island | Namatakula,  Fiji | Suva, Fiji | Réunion  Island | Madagascar | Kiribati | Keeling  Islands | Seychelles | Taiwan | Guam |
| --- | --- | --- | --- | --- | --- | --- | --- | --- | --- | --- | --- | --- | --- |
| Kingman Reef | -0,059 |  |  |  |  |  |  |  |  |  |  |  |  |
| Isla del Coco | 0.087* | 0.008 |  |  |  |  |  |  |  |  |  |  |  |
| Clipperton Island | -0.034 | -0.031 | 0.094 |  |  |  |  |  |  |  |  |  |  |
| Namatakula, Fiji | 0.039 | 0.032 | 0.057 | 0.453* |  |  |  |  |  |  |  |  |  |
| Suva_Fiji | 0.032 | -0.049 | -0.075 | 0.066 | 0.050 |  |  |  |  |  |  |  |  |
| Réunion Island | 0.141* | 0.031 | -0.075 | 0.139 | 0.073 | -0.099 |  |  |  |  |  |  |  |
| Madagascar | -0.035 | -0.073 | 0.009 | -0.031 | 0.007 | -0.063 | -0.009 |  |  |  |  |  |  |
| Kiribati | -0.033 | -0.071 | 0.064 | -0.045 | 0.073 | 0.003 | 0.103 | -0.049 |  |  |  |  |  |
| Keeling Islands | 0.092* | -0.009 | -0.049 | 0.160 | 0.099 | -0.077 | -0.052 | 0.019 | 0.073 |  |  |  |  |
| Seychelles | -0.006 | -0.060 | -0.025 | 0.022 | -0.031 | -0.085 | -0.037 | -0.063 | -0.014 | -0.035 |  |  |  |
| Taiwan | -0.098 | -0.117 | 0.032 | <0.000 | 0.322 | -0.031 | 0.044 | -0.117 | -0.121 | 0.069 | -0.058 |  |  |
| Guam | -0.070 | -0.122 | -0.011 | -0.018 | 0.035 | -0.072 | 0.007 | -0.087 | -0.084 | -0.031 | -0.080 | -0.109 |  |
| Marshall Islands | -0.067 | -0.088 | 0.033 | 0.152 | 0.267 | -0.022 | 0.041 | -0.096 | -0.083 | 0.076 | -0.043 | <0.000 | -0.081 |
